# The hidden emotional costs of giving life: preserving donors’ mental health and quality of life after living kidney donation

**DOI:** 10.1007/s40620-025-02291-9

**Published:** 2025-04-19

**Authors:** Xavier Torres, David Paredes-Zapata, Ignacio Revuelta

**Affiliations:** 1 Clinical Health Psychology Section, Psychiatry and Clinical Psychology Service, Clinic Institute of Neurosciences, Hospital Clinic, Villarroel 170, 08036 Barcelona, Spain; 2 Departament de Cirurgia i Especialitats Medicoquirúrgiques, Facultat de Medicina i Ciències de la Salut, Universitat de Barcelona, Barcelona, Spain; 3 Donation and Transplant Coordination Section, Hospital Clinic, Villarroel 170, 08036 Barcelona, Spain; 4 Surgical and Medical Specialties Department, University of Barcelona, Barcelona, Spain; 5 Donation and Transplantation Institute (DTI) Foundation, Barcelona, Spain; 6 Kidney Transplant Unit, Department of Nephrology and Renal Transplantation, Hospital Clinic, Villarroel 170, 08036 Barcelona, Spain; 7 Surgical and Medical Specialties Department, University of Barcelona, Barcelona, Spain; 8 Red de Investigación Renal (RICORS), Madrid, Spain; 9 Kidney Transplant Group (SENTRA), and Bid Data Working Group (BIGSEN), Spanish Society of Nephrology (SEN), Madrid, Spain

**Keywords:** Kidney living donor, Kidney transplantation, Quality of life, Psychological evaluation, Psychosocial evaluation, Psychological vulnerabilities

## Abstract

Kidney living donation remains the best treatment available for kidney failure. Most living donors report positive personal outcomes, such as enhanced life satisfaction and personal growth. However, mental health challenges have also been documented. The study by Tahir, Aftab and Nabi (J Nephrol 10.1007/s40620-025-02217-5, 2025) call the attention to a small subset of living donors who may experience significant depression symptoms and occasionally suicidal ideation after donation, particularly when the recipient dies or suffers severe graft failure with a return to dialysis. As observed in the previous studies, only donors whose recipients experienced negative outcomes reported mood alterations or life dissatisfaction (Menjivar et al., Transpl Int 31(12):1332–1344, 2018). These rare post-donation risk scenarios justify a careful mental evaluation to identify psychological vulnerabilities or a history of difficulties in managing and coping with stressful situations. These adverse outcomes appear more likely in donors with pre-donation physical and/or psychological vulnerabilities, in those with a complicated surgical recovery after donation, and in cases where recipients experience poor physical or psychological outcomes. Moreover, cases of graft failure or recipient death might significantly increase donor's likelihood of depression and anxiety, Despite the generally low incidence of psychosocial problems after donation, there have been calls for a more structured and routine follow-up assessment to further mitigate risks and ensure equitable mental health safety for all kidney donors.

Kidney living donation remains the best treatment available for kidney failure. Most living donors report positive personal outcomes, such as enhanced life satisfaction and personal growth. However, mental health challenges have also been documented.

The study by Tahir, Aftab and Nabi [1] reported low incidence of mental health problems after donation—only 9% of donors reported depressive symptoms—and a high level of satisfaction, with only 8% expressing dissatisfaction with life after donation, and only slightly so. As observed in previous studies, only donors whose recipients experienced negative outcomes reported mood alterations or life dissatisfaction [2]. Along the same line, the study by Tahir, Aftab and Nabi [1] call attention to a small subset of living donors who may experience significant depression symptoms and occasionally suicidal ideation after donation, particularly when the recipient dies or suffers severe graft failure with a return to dialysis. These rare post donation scenarios justify careful mental evaluation to identify psychological vulnerabilities or a history of difficulties in managing and coping with stressful situations. This need is especially critical in societies where women face complex and fragile socioeconomic conditions, particularly when the donor is dependent on the recipient and the partner is at high risk of graft failure or death after transplantation.

Despite the generally low incidence of psychosocial problems after donation, there have been calls for a more structured and routine follow-up assessment to further mitigate risks and ensure equitable mental health safety for all kidney donors. Certainly, depression rates among donors can range from 0 to 46.9%, with anxiety rates reaching as high as 66.7%. Other adverse outcomes—such as donors’ worsening quality of life, diminished life satisfaction, and regret after donation—have been reported. These adverse outcomes appear more likely in donors with pre-donation physical and/or psychological vulnerabilities, in those with a complicated surgical recovery after donation, and in cases where recipients experience poor physical or psychological outcomes [3, 4]. Moreover, the most severe outcome after donation—namely, the death of the recipient—triples the risk of donors disengaging from follow-up care and might significantly increase their likelihood of depression and anxiety [5]. Graft failure similarly increases the risk of poorer mental health, especially among family donors, whose emotional investment is often greater [6]. Family donors may also experience strained relationships, highlighting the complex psychological facets of donation, even in successful transplants [7]. These emotional risks are further complicated by socioeconomic variables. Financial hardships, including direct and indirect costs of donation, disproportionately affect donors in resource-constrained settings, and may exacerbate the risk of adverse psychosocial outcomes [8, 9].

It seems surprising that, although Tahir, Aftab and Nabi’s findings confirm those observed in numerous previous studies, a robust follow-up framework of living kidney donors remains unusual in clinical practice. It seems particularly relevant given that one of the main complaints of living donors—and a primary source of dissatisfaction with donation—is the significant decrease in the amount of care received from healthcare professionals after donation. Providing better information during the evaluation process for donation could help address these gaps in donors’ expectations. Additionally, given the low overall incidence of psychosocial issues among donors after donation, a potential strategy to optimize post-donation follow-up costs might involve defining the specific cases in which such follow-up is advisable.

Recommendations drawn from previous findings might help avoid unnecessary or unwanted follow-up assessments (see Table 1).

**Table 1:** Summary of follow-up recommendations

Study references	Main findings	Recommendations for follow-up
Torres et al. 2017 [4]	Recipient death triples donor drop-out risk and probably the risk of psychological distress	Prioritize follow-up for donors after adverse recipient outcomes
Menjivar et al. 2018 [2]	Only donors whose recipients experienced negative outcomes reported mood alterations or life dissatisfaction	Assess social support and prioritize donor follow-up mainly in those whose recipients experienced negative outcomes
Davidson et al. 2019 [8]	Limited healthcare resources exacerbate donor challenges in low-resource countries	Implement telemedicine and financial aid programs in resource-poor settings
Timmerman et al. 2016 [6]	Lack of social support predicts poor mental health outcomes post-donation	Assess social support and decide for including its improvement as part of follow-up care Enhance social integration and community support for donors
Ong et al., 2021 [3]	Depression and anxiety are common post-donation in vulnerable groups	Provide targeted early psychological interventions for high-risk donors
Clarke et al., 2006 [7]	Economic costs disproportionately burden donors in lower-income settings	Develop reimbursement policies for direct and indirect donation expenses
Cazauvieilh et al. 2023 [4]	Family-related donations carry unique psychological complexities	Offer family counseling and relational support post-donation in high-risk scenarios

It is crucial to conduct a thorough psychological screening and provide education about potential risks. Evaluating non-realistic expectations (such as the complete absence of pain after surgery), pre-existing mental health conditions, certain coping styles, and the donor-recipient relationship is essential to identify donors at risk for adverse psychosocial outcomes [2, 7].

Recipient death or physical adversities—including, but not limited to, graft complications or failure—should be regarded as high-risk scenarios and, as such, serve as red flags for the need to refer donors for follow-up mental health assessments. Special attention should be given to the assessment of the emotional and social well-being of family donors and those from low-resource settings, even if this approach increases sensitivity at the expense of specificity [8, 9].

Targeted interventions, peer support groups and prompt financial assistance for at-risk donors may help mitigate psychosocial risks and potentially prevent the need for longer and more costly interventions. Telemedicine services could also provide accessible solutions, especially in resource-limited countries [8].

Governments, international organizations and transplant centers might consider integrating psychological donor follow-up into healthcare strategies to ensure that all at-risk donors, regardless of economic background, have access to comprehensive post-donation care [9]. In this sense, it is valuable to refer to published Guidelines from International Scientific Societies, such as the International Society of Nephrology, relative to Living Donor Evaluation and Follow-up.

In summary, living donation exemplifies altruism and compassion; however, the mental health of donors should not be overlooked due to the success of the transplant. Structured follow-up programs that address psychological, social, and financial challenges are necessary to safeguard donors' well-being, particularly when high-risk scenarios are identified. This is especially important in resource-limited settings, where disparities may further amplify risks.

## Data Availability

Not applicable.
